# Uncertainty Quantification in Zip Code Tabulation Area-Level Breast Cancer Screening: A Bayesian Geospatial Analysis in Hillsborough County, Florida

**DOI:** 10.3390/ijerph23070911

**Published:** 2026-07-16

**Authors:** Bhaveshsai Reddy, Aarya Satardekar, Namit Choudhari, Benjamin G. Jacob, Rishil Shah, Anusha Parajuli

**Affiliations:** 1 College of Arts and Sciences, University of South Florida, Tampa, FL 33620, USA; 2 College of Public Health, University of South Florida, Tampa, FL 33620, USA; aaryasatardekar9@gmail.com (A.S.); bjacob1@usf.edu (B.G.J.); anushaparajuli@usf.edu (A.P.); 3 School of Geosciences, University of South Florida, Tampa, FL 33620, USA; nchoudhari@usf.edu; 4 Bellini College of Artificial Intelligence, Cybersecurity and Computing, University of South Florida, Tampa, FL 33620, USA; rshah10@usf.edu

**Keywords:** spatial epidemiology, Bayesian regression, Poisson model, negative binomial model, Moran’s I, health disparities, small-area analysis, geospatial modeling, breast cancer screening, ZIP Code Tabulation Area

## Abstract

**Highlights:**

**Public health relevance—How does this work relate to a public health issue?**

- Geographic disparities in breast cancer screening continue to drive late-stage diagnosis and excess mortality, particularly among minoritized women living in demographically heterogeneous U.S. counties.
- Small-area surveillance at the ZIP Code Tabulation Area (ZCTA) scale, combined with explicit uncertainty quantification, can localize where screening activity is concentrated and where targeted public health resources are most needed.

**Public health significance—Why is this work of significance to public health?**

- This study applies a Bayesian geospatial framework with Jeffreys’ non-informative priors—an underused approach in breast cancer surveillance—to quantify uncertainty in ZCTA-level screening counts using Markov Chain Monte Carlo estimation.
- Breast cancer screening exam counts were significantly related to the number of females in each race/ethnic group. Median household income and health insurance coverage were not independent predictors in the multivariate models.

**Public health implications—What are the key implications or messages for practitioners, policy makers and/or researchers in public health?**

- Screening surveillance based on point estimates alone may misrepresent disparities; posterior credible intervals are needed to flag ZCTAs where uncertainty itself signals data or service gaps.
- Demographically informed strategies—mobile mammography, patient navigation, and culturally responsive outreach—are more appropriate than adjacency-based geographic targeting in counties where screening activity varies by demographic concentration rather than by spatial diffusion.

**Abstract:**

Geographic variation (GV) in screening patterns for breast cancer exists among Zip Code Tabulation Areas (ZCTAs) in Florida, but most spatial analyses are based on frequentist point estimates which do not formally represent uncertainty. This study used a three-stage analytical approach to the breast cancer screening data at the zip code tract (ZCTA) level in Hillsborough County, Florida (*n* = 55 ZCTAs): first, a frequentist Poisson regression was applied with diagnostics for multicollinearity; second, global spatial autocorrelation (GSA) analysis was conducted using Moran’s I; and third, a Bayesian Poisson and a Bayesian negative binomial regression were performed, both estimated via the No-U-Turn Sampler in the brms/Stan framework. In ArcGIS Pro 3.6, spatial analyses were carried out. The dependent variable was the number of breast cancer screening exams conducted at the ZCTA level over the study period. Across all racial/ethnic subgroups, the observed number of screenings was correlated with the number of females in the household, while no independent correlation was found for median household income, insurance status or age by stratum variables after adjustment. There was no significant and strong spatial autocorrelation across the study area (Moran’s I = 0.003, z = 0.326, *p* = 0.745). The Poisson model did best among the Bayesian models with a Bayesian R^2^ of 0.91, RMSE of 5.40, and MBE of 0.02. The results show the usefulness of Bayesian uncertainty quantification in small area public health surveillance and offer a framework for quantifying geographic variation in screening activity in a probabilistic manner. The results only compare screening examination (not population-standardized screening rates) and should be considered to reflect screening volume rather than screening participation.

## 1. Introduction

Breast cancer remains one of the most commonly diagnosed cancers among women worldwide and a leading cause of cancer-related mortality [1,2]. In the United States, approximately one in eight women will develop breast cancer over their lifetime, underscoring its substantial public health burden [3,4]. Florida reflects this national pattern while also exhibiting pronounced geographic variation, reporting more than 20,000 new female breast cancer cases annually at incidence rates comparable to national averages [5]. Screening and mortality outcomes vary substantially across counties and communities, influenced by differences in early detection, access to care, and underlying social and structural conditions. Despite comparable or higher reported screening rates in some groups, non-Hispanic Black women continue to experience disproportionately higher mortality, highlighting that factors beyond screening participation—such as delayed diagnosis, access disparities, and systemic inequities—play important roles in outcomes [3,6].

Mammographic screening has been associated with substantial reductions in breast cancer mortality in randomized trials and population-level modeling analyses [7,8], although the optimal balance of benefits and harms continues to be debated [9]. National monitoring continues to document widening incidence–mortality gaps between racial and ethnic subgroups [4,10], driven by intersecting structural, biological, and access-related factors [10,11]. The COVID-19 pandemic substantially disrupted breast cancer screening services, producing measurable and uneven shortfalls in mammography utilization that disproportionately affected minoritized populations and persisted well after the initial pandemic wave [12,13,14]. These contemporary trends reinforce the need for small-area surveillance that can localize where screening activity is concentrated, where it has eroded, and where uncertainty in the underlying counts is greatest.

These disparities are evident within Florida, where socioeconomically underserved areas experience worse outcomes and later-stage diagnoses [5]. Geographic factors, including accessibility to screening facilities, travel time to diagnostic and treatment centers, neighborhood deprivation, and local healthcare infrastructure, influence access to care and the timeliness of diagnosis [15,16,17]. Evidence further indicates that women in rural or peripheral regions often experience worse survival outcomes than their urban counterparts, even after adjustment for clinical stage and treatment receipt [15]. Collectively, these patterns suggest that breast cancer vulnerability is spatially heterogeneous, forming localized regions of elevated or reduced risk shaped by structural, demographic, and environmental conditions. Statewide or aggregate-level summaries, therefore, tend to obscure sub-regional variation that is critical for targeted public health planning. Neighborhood-level socioeconomic context is now widely recognized as an independent determinant of cancer outcomes [18], and limited geographic access to mammography facilities has been associated with later-stage diagnosis and reduced screening utilization across the United States [19].

Although geospatial methods for cancer screening surveillance have advanced rapidly, most published ZCTA- or census-tract-level analyses still rely on frequentist point estimation [20,21]. Frequentist estimators do not formally quantify the uncertainty associated with each small-area estimate, which is particularly limiting in counties with a moderate number of ZCTAs where asymptotic inference can be unreliable [22,23,24]. Bayesian disease mapping methods address this gap by treating each ZCTA-level coefficient and predictive count as a posterior distribution, yielding credible intervals that integrate both data variability and prior uncertainty [23,24,25]. When non-informative priors such as the Jeffreys prior are used, the resulting posterior inference is data-driven and comparable to maximum-likelihood estimation while still providing a complete characterization of uncertainty [23,26].

The current research, therefore, seeks to fill in some of these gaps by incorporating an uncertainty-focused Bayesian geospatial framework in examining the breast cancer screening examinations at ZCTA level in Hillsborough County, Florida. While small area studies conducted in the past have concentrated mainly on determining the factors that correlate with the number of breast cancer screenings done within specific small areas using classical point estimate techniques such as frequentist approaches, there seems to be insufficient work conducted on determining the uncertainty that surrounds screening counts and model predictions. This analytical framework combines the frequentist approach to Poisson regression modeling, test for spatial autocorrelation using Moran’s I, and Bayesian count regression models through MCMC techniques with Jeffreys non-informative priors. The aim is not just to examine the variation in screening examination counts across ZCTAs but also the uncertainty that accompanies these numbers.

## 2. Materials and Methods

### 2.1. Study Area and Data Sources

The study was conducted in Hillsborough County, Florida, USA. Hillsborough County is the fourth most populous county in Florida, encompassing the Tampa metropolitan area and a diverse mix of urban, suburban, and semi-rural communities. It presents an analytically useful setting for ZCTA-level breast cancer research, given its demographic heterogeneity, documented variation in cancer screening outcomes, and the availability of spatially referenced public health data [5]. Area-based analyses at the ZIP-code or ZCTA scale provide a policy-relevant geographic resolution for monitoring socioeconomic and racial inequities in cancer screening [27].

The unit of observation is the ZIP Code Tabulation Area (ZCTA), which is a geographically bounded entity developed by the U.S. Census Bureau as an approximation of ZIP code boundaries for statistical purposes. The sample consisted of *n* = 55 observations, where each ZCTA i = 1, 2, …, *n* represented a geographical spatial unit. For each ZCTA i, the covariate vector Xi includes demographic, socio-economic, and insurance-related data variables, each one collected at the ZIP code level.

The outcome variable was ZCTA-level breast cancer screening counts (CASES), or the number of breast cancer screening examinations that were ever recorded in any given geographic unit during the study period. CASES represents aggregated breast cancer screening examinations recorded at the ZCTA level and does not represent screening rates or unique individuals. These data were secondary, aggregated data and were only available at the ZCTA level. The data set was not designed for the ability to identify individual subjects, differentiate first and repeat screening examinations or connect to individual clinical data. Therefore, the data for CASES should not be used to count the number of women screened in each ZCTA but should be used as the volume of screening activity that has occurred. However, population-standardized screening rates and denominator data on eligible screening populations were not available, so the analysis looks at geographic variation in screening examination counts rather than screening participation rates.

Covariates were drawn from publicly available US Census Bureau and public health data sources. They included racial and ethnic female population counts (White, Black or African American, Hispanic or Latino, Asian, and American Indian or Alaska Native), age-stratified female population counts (40–49, 50–64, and 65 and older), median household income, and the uninsured female population count. Table 1 summarizes all variables. Spatial coordinates and geographic boundary files were incorporated to support spatial autocorrelation analyses.

### 2.2. Analytical Framework

The analysis was performed in three stages: (1) frequentist Poisson regression analysis to determine association between ZCTA-level demographic, socioeconomic and insurance-related characteristics with breast cancer screening examination counts and assess model assumptions, (2) global spatial autocorrelation analysis to evaluate whether there was evidence of spatial dependence in screening activity across ZCTAs and (3) Bayesian count regression analysis to quantify uncertainty about model parameters and compare alternative model specifications. This analytical approach takes an uncertainty-oriented view, using frequency-based methods (the frequentist approach) and adding to them with Bayesian posterior inference, giving a more complete picture of geographic variation in breast cancer screening activity. The framework combines the statistical association analysis, spatial evaluation, and uncertainty quantification approaches to aid in the interpretation of small-area screening patterns.

### 2.3. Frequentist Regression Modeling

#### 2.3.1. Poisson Regression

Poisson regression was employed as the primary count model, implemented in R. The dependent variable Y_i_ was assumed to follow a Poisson distribution. Regression coefficients were estimated using maximum likelihood. Multicollinearity was assessed using variance inflation factors (VIFs) computed from the design-matrix inverse [28]; variables exceeding conventional thresholds were removed stepwise. Overdispersion was evaluated using a formal dispersion test, and the goodness-of-fit was assessed using deviance residuals and the ratio of residual deviance to degrees of freedom.

#### 2.3.2. Negative Binomial Regression

A frequentist negative binomial model was estimated in R as a robustness check to verify that any latent overdispersion not captured by formal testing did not alter the magnitude or direction of the coefficient estimates.

### 2.4. Spatial Autocorrelation Analysis

Global spatial autocorrelation was quantified using Moran’s I [29], implemented in ArcGIS Pro with inverse-distance weighting and Euclidean distance applied to point-referenced ZCTA data. The expected value of I under complete spatial randomness is −1/(*n* − 1); deviations from this value were evaluated using a standardized z-score under a randomization null model [30,31]. A two-sided *p*-value below 0.05 was treated as evidence against the null hypothesis of spatial randomness.

### 2.5. Bayesian Count Regression

The Bayesian Poisson regression and Bayesian negative binomial models were estimated in R using brms package [32], whose underlying sampling engine was Stan. Jeffreys’ non-informative priors were employed in order to provide the objectivity and prior-parameterization invariance inherent in the Jeffreys’ framework. Specifically, the use of Jeffreys priors in our Bayesian analyses was justified given the exploratory nature of the study, lack of previous evidence pertaining to the parameter value inferences at ZCTA level, and desire to quantify uncertainty without introducing bias that could be attributed to a particular choice of the prior. This enabled us to obtain posterior inference mostly based on the data, but to additionally derive credible intervals and probability measures of uncertainty that cannot be derived using the classical maximum likelihood methods. Posteriors were estimated using No-U-Turn Sampler (NUTS), which is a variant of the Hamiltonian Monte Carlo sampler, designed to efficiently explore complex posterior distributions. Details on the MCMC configuration are provided in Table 2 below. The convergence of the chains was diagnosed via the Gelman-Rubin potential scale reduction factor (R̂) test, which was deemed to have converged when R̂ ≤ 1.01 along with bulk and tail effective sample size criteria [26]. Conditional autoregressive models with hierarchical structures, including the Besag-York-Mollie (BYM) approach that leverages strength from neighbors, were examined [25] but found no use since no statistically significant global spatial autocorrelation could be detected (see Section 3.4).

#### Model Comparison

Bayesian Poisson and Bayesian Negative Binomial models were compared using the Deviance Information Criterion (DIC), Bayesian R^2^, Root Mean Square Error (RMSE), and Mean Bias Error (MBE) between posterior predictive means and observed counts. Posterior predictive checks were conducted to evaluate distributional fit, including comparisons of replicated and observed count distributions across the full range of ZCTAs.

### 2.6. Ethical Considerations, Generative AI, and Data Availability

This study used aggregated, publicly available ZCTA-level data and did not involve individual-level human subjects. Institutional review board approval was therefore not required. Generative AI-assisted tools were used only for grammar checking, language editing, and sentence-level clarity. No generative AI tools were used to generate the study design, data, analyses, figures, tables, results, interpretation, or conclusions. All authors reviewed and verified the final manuscript content and take full responsibility for the work. All data, model specifications, and R code will be made available upon reasonable request to the corresponding author.

## 3. Results

### 3.1. Descriptive Overview of Breast Cancer Screening Counts

Breast cancer screening counts varied substantially across ZCTAs in Hillsborough County, Florida, ranging from 3 to 100 cases. Several ZCTAs exhibited markedly higher counts relative to surrounding areas, while others recorded consistently lower counts. Screening activity was not uniformly distributed across the study region.

### 3.2. Frequentist Poisson Regression Results

Initial Poisson regression specifications incorporating total population, age-stratified female population variables, racial and ethnic composition, insurance coverage, and median household income exhibited multicollinearity, with VIFs exceeding acceptable thresholds. Following stepwise removal of total population and correlated age variables, all remaining VIFs fell below conventional thresholds. The dispersion statistic was 0.87 (*p* = 0.61), supporting Poisson specification.

The results show that the demographic profile of ZCTAs is significantly related to screening volume measures. Nonetheless, since the model accounts for screening counts and not population-based standardized measures of screening rate, some of the observed associations may be partially driven by population size and demographic concentrations across locations. Female population counts among White, Black or African American, and Hispanic or Latino groups were each statistically significant (Table 3). Median household income did not reach significance. Age-group variables were not significant in adjusted models. The Hispanic or Latino female population variable was significant once age strata were removed. A negative binomial robustness check yielded a very large dispersion parameter (θ = 622,590) with coefficient estimates and significance patterns unchanged.

### 3.3. Spatial Visualization and Hot/Cold Spot Patterns

**Figure 1:**
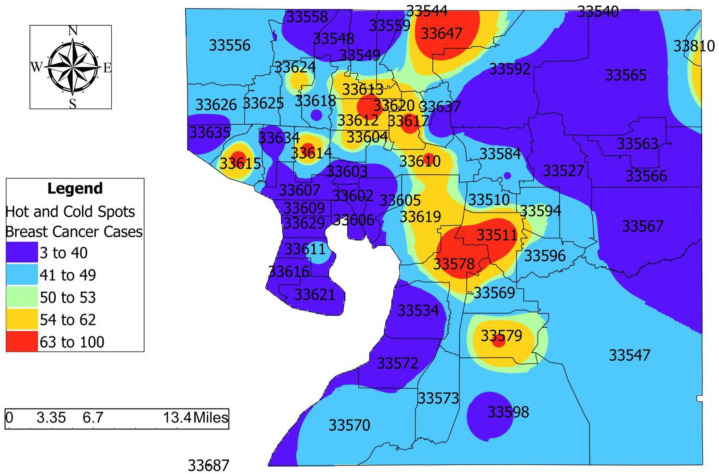
Hot- and cold-spot kernel density heatmap of breast cancer screening counts by ZCTA in Hillsborough County, Florida. Generated in ArcGIS Pro using inverse-distance weighted interpolation. High-count ZCTAs (63–100) concentrate in central and northeastern areas; low-count ZCTAs (3–40) appear in peripheral and western portions of the county.

Spatial visualization of ZCTA-level screening counts identified localized areas of elevated intensity across Hillsborough County, following established choropleth- and atlas-mapping conventions for public-health surveillance [33]. Several ZCTAs recorded high screening counts (63–100 cases), concentrated primarily in the central and northeastern portions of the county. Cold-spot ZCTAs (3–40 cases) were observed predominantly in peripheral and western areas (Figure 1).

### 3.4. Global Spatial Autocorrelation (Moran’s I)

Global spatial autocorrelation analysis yielded a Moran’s Index of 0.002727, with an expected index of −0.019608 under complete spatial randomness. The associated z-score was 0.325651 and the *p*-value was 0.744688 (Table 4). The null hypothesis of complete spatial randomness was therefore not rejected.

### 3.5. Bayesian Count Regression Results

The Bayesian Poisson model converged fully across all four chains following burn-in, with R̂ = 1.00 for all parameters and adequate bulk and tail effective sample sizes (Table 5). Posterior predictive checks confirmed adequate distributional fit. The three demographic predictors were confirmed: Female_Population_White (β = 0.22, 95% CrI: 0.15–0.28), Female_Black_African_American (β = 0.13, 95% CrI: 0.06–0.19), and Female_Population_Hispanic_Latino (β = 0.09, 95% CrI: 0.00–0.19). All remaining predictors had credible intervals spanning zero. The Bayesian Negative Binomial model yielded similar coefficient directions but wider credible intervals for all predictors, with the shape parameter estimated at θ = 12.52 (95% CrI: 7.76–18.60).

Model comparison favored the Bayesian Poisson specification across all four criteria (Table 6). The Bayesian Poisson achieved Bayesian R^2^ = 0.91 (SE: 0.01), RMSE = 5.40, MBE = 0.02, and DIC = 367.2, compared with R^2^ = 0.82 (SE: 0.04), RMSE = 8.31, MBE = 1.40, and DIC = 409.4 for the Bayesian Negative Binomial.

## 4. Discussion

### 4.1. Demographic Composition and Screening Volume

The most consistent result of this study was that observed counts of breast cancer screening examinations in the ZCTAs were consistently correlated with female population counts, regardless of racial/ethnic group, for the county of Hillsborough. The associations were found across a number of frequentist Poisson model specifications and were quantified with Bayesian posterior estimation. The results presented, however, are based on the number of screening examinations conducted and do not reflect population-based screening rates and should be interpreted with caution. Geographic differences in the number of female to male residents would be expected to lead to different numbers of screenings, so the associations observed may be due to demographic differences as well as to screening differences. The Bayesian approach is key not just in identifying demographic associations but also in formally quantifying uncertainty about these associations with posterior distributions and credible intervals. The Black or African American finding is important to be interpreted in conjunction with the following note: A high screening rate should not be viewed as an indicator of equity in screening services in ZCTAs where there is a large Black female population. Although there are no major differences in screening rates, Black women in the United States have significantly higher breast cancer mortality rates, which may be due to a combination of diagnostic delays, tumor biology, healthcare access barriers, and systemic inequities [6,34,35]. Aggregate ZCTA screening counts reflect neither screening uptake nor timeliness of follow-up after an abnormal mammogram, stage at diagnosis, nor quality of follow-up care. This means that the high density of screening activity in ZCTAs with high percentages of Black female residents is more likely a measure of the demographics of the residents than of the quality of care.

### 4.2. Non-Significance of Income, Insurance, and Age

Median household income, insurance status, and age-stratified population variables were not significantly associated variables in any stable model specification. At the ZCTA level, racial and ethnic composition absorbs most of the between-area variance in screening counts, leaving income and insurance with little independent explanatory power, a pattern consistent with ecological analyses of cancer screening in heterogeneous urban counties [36,37]. Age is well-established as a critical individual-level risk factor for breast cancer [38], but at the ZCTA scale, its explanatory contribution is attenuated because screening infrastructure is already calibrated to age-based eligibility criteria, and because age distribution is correlated with racial/ethnic composition in demographically diverse areas. The attenuation of the Hispanic or Latino coefficient in age-adjusted models reflects this shared variance and was resolved through stepwise VIF-based covariate selection.

### 4.3. Spatial Heterogeneity Without Global Spatial Dependence

The Moran’s I value for breast cancer screening examination counts was 0.003 (z = 0.326, *p* = 0.745), indicating that the counts were geographically heterogeneous, but not statistically significantly spatially autocorrelated globally across ZCTAs in Hillsborough County. Because global Moran’s I did not indicate significant spatial dependence, local cluster analyses such as LISA and Getis-Ord Gi* were not pursued. The geography of screening activity observed is therefore more likely to reflect local demographic features than widespread screening spatial clustering processes [30,39]. The lack of global spatial autocorrelation does not detract from the usefulness of spatial analysis in public health surveillance, importantly. Instead, it implies that screening activity does not occur via a global Moran’s I statistic or large-scale spatial diffusion processes. Spatial methods are still useful as they help identify geographic heterogeneity, map the local screening patterns, and evaluate the variation in the demographic/socioeconomic distribution of populations across geographic areas. Therefore, the present results should not be interpreted as saying geography doesn’t matter, but rather that geography was not broadly clustered at the ZCTA level in the study area. This analysis could be extended in the future by the use of Local Indicators of Spatial Association (LISA) statistics, Getis–Ord Gi* statistics, and other local spatial clustering methods that can detect localized hot spots and cold spots that are not detected by the global measures alone [30,40]. Furthermore, population-adjusted spatial statistics should be used to assess the robustness of spatial patterns in the presence of different population densities and screening volumes in ZCTAs [31,41].

### 4.4. Value of the Bayesian Framework

The Bayesian Poisson model substantially outperformed the Bayesian Negative Binomial across all comparison criteria: Bayesian R^2^ = 0.91 vs. 0.82, RMSE = 5.40 vs. 8.31, MBE = 0.02 vs. 1.40, and DIC = 367.2 vs. 409.4. The Jeffreys prior produced calibrated posterior inference without imposing strong prior assumptions, and full convergence (all R̂ = 1.00) was confirmed across all parameters. Beyond model fit, the Bayesian framework provided formal credible intervals for each ZCTA-level coefficient, an advantage over frequentist point estimates in a small-area analysis with *n* = 55 ZCTAs, where asymptotic inference can be unreliable [22,23,24]. ZCTAs with wide credible intervals should be treated as priorities for data collection as well as potential intervention, as their classifications carry greater uncertainty.

### 4.5. Public Health Implications

At the public health level, the results of the current study could contribute to the discussion of a demographically informed breast cancer screening surveillance and resource allocation based on equity. The results should not be taken as evidence of disparities in screening participation, access, or healthcare utilization because the analysis was conducted using counts of screening examinations per examination rather than screening rates per population. Rather, the findings pinpoint geographic areas that show variability in screening activity levels and the uncertainty that exists around those activities across ZCTAs. The results from the demographic associations, the spatial analysis, and the Bayesian uncertainty quantification suggest that geographic variation in screening activity could be explored further in settings with high screening volume, low screening volume, and/or significant uncertainty in the number of screenings observed. There is existing public health literature that suggests that patient navigation programs [35,42], mobile mammography services [43], community health worker outreach [44], and structured follow-up systems for abnormal mammograms [45] can increase the access and diagnostic resolution of underserved populations to screening and/or diagnosis. The present study is not designed to assess the effectiveness of these interventions, but the results of this study may be useful to help identify geographic areas for additional assessment/surveillance/targeted evaluation to consider. Another benefit of the Bayesian approach is that it allows for the measurement of uncertainty in small areas. ZCTAs that showed higher degrees of posterior uncertainty may be regions where further data collection, monitoring, or community-level evaluation might lead to a better understanding of screening rates at the community level. Therefore, uncertainty estimates can be used as a secondary tool to aid in evidence-informed public health planning and future research priorities.

### 4.6. Limitations

A major constraint of this study is that the response variable was assessed using the total number of breast cancer screening exams documented instead of the population-standardized screening rate. The reason for this is that the denominator for estimating the eligible population was unavailable, thus making it impossible to differentiate between increased screening and increased population. Some of the relationships found can be a result of this population aggregation rather than differences in screening behavior. Future studies need to consider incorporating population-standardized screening rates and using offset variables in count models.

There are other limitations with this study as well. One is that the use of an ecological design makes individual inference not possible; associations found at the ZCTA level should not be extrapolated to individual women [29]. The second limitation is that Moran’s I statistic may not be able to detect local spatial autocorrelation and may be biased because of differences in population size between ZCTAs [31,41]. Third, the smaller number of observations made on ZCTAs (*n* = 55) restricts what types of models may be estimated reliably. The predictor variable set is limited only to census-based variables, which means that some relevant predictors like facility access measures, physician densities, barriers to transportation, health literacy, and healthcare utilization attributes could not be included. The final limitation of using cross-sectional data is that there will be no way to examine any temporal patterns and assess causation. There will also be an inability to observe the effects of new policies, like the change to the mammography screening recommendations for 2024 by the USPSTF that lowered the age recommendation from 50 to 40 [3].

## 5. Conclusions

This research used a holistic uncertainty-based methodological approach that consisted of classical Poisson regression modeling, global spatial autocorrelation measures, and Bayesian count regression modeling to analyze the geographic variability in the number of women who had breast cancer screening examinations across ZCTAs in Hillsborough County, Florida. These results show the importance of using Bayesian uncertainty analysis in public health surveillance of small areas and suggest the possibility of applying probabilistic approaches to evidence-based decision-making. The number of females within various major racial and ethnic groupings was found to correlate significantly with breast cancer screening examination counts. In contrast, factors such as income level, insurance, and age-stratified variables showed no significant association with breast cancer screening counts when the models were statistically sound. Given that the outcome variable was breast cancer screening examination counts rather than rates, this finding must be considered as being indicative of variability in the level of screening performed rather than in the level of screening participation. Global spatial autocorrelation was very low, indicating a lack of a spatially coordinated pattern. The findings suggest that it is less likely that spatial diffusion plays a role in creating geographical variations as opposed to local demographic factors.

In terms of the Bayesian methods that were tested, the Bayesian Poisson regression performed very well, providing credible intervals that quantified the uncertainty surrounding the parameter estimates of the model. The most important contribution of this study is not just the investigation of geographic variations in the utilization of breast cancer screenings, but also an illustration of the power of Bayesian uncertainty quantification methodology to use in public health studies involving small area data. Future studies should adopt standardized screening rates for the population, local cluster analyses, and Bayesian disease mapping hierarchies.

## Figures and Tables

**Table 1:** ZCTA-Level Variables Included in the Analysis, Hillsborough County, Florida.

Category	Variable	Description
Outcome	CASES	Total recorded breast cancer screening examination counts within each ZCTA during the study period (aggregated screening activity; not unique individuals or population-standardized screening rates)
Racial/Ethnic Composition	Female_Population_White	White female population count
	Female_Black_African_American	Black or African American female population count
	Female_Population_Hispanic_Latino	Hispanic or Latino female population count
	Female_Population_Asian	Asian female population count
	Female_Population_American_Indian_Alaska	American Indian or Alaska Native female population count
Age Strata	Female_40_49	Female population aged 40–49 years
	Female_50_64	Female population aged 50–64 years
	Female_65	Female population aged 65 years and older
Socioeconomic	Median_Income	Median household income (USD)
Insurance	Female_Uninsured	Uninsured female population count

Note. All population variables are totals of females at the ZCTA level. CASES is the number of breast cancer screening exams that were recorded in each ZCTA over the course of the study. Data available did not allow for identification of individual patients or differentiating between first and repeat screening examinations. N = 55 ZCTAs; ZCTA = ZIP Code Tabulation Area.

**Table 2:** MCMC Sampling Configuration for Bayesian Regression Models.

Model Specification	Chains	Iterations	Warmup	Post-Warmup Draws	Sampler
Bayesian Poisson	4	4000	2000	8000	NUTS
Bayesian Negative Binomial	2	2000	1000	2000	NUTS

**Table 3:** Final Reduced Frequentist Poisson Regression Model: Associations with Breast Cancer Screening Examination Counts by ZCTA, Hillsborough County, Florida.

Variable	Estimate (β)	Std. Error	z-Value	*p*-Value
White female population	0.0000312	0.0000041	7.61	<0.001
Black/African American female population	0.0000897	0.0000112	8.01	<0.001
Hispanic or Latino female population	0.0000584	0.0000143	4.08	<0.001
Median household income	0.0000021	0.0000018	1.17	0.243
(Intercept)	3.641	0.187	19.47	<0.001

Note. VIF < 5.0 for all retained predictors. Dispersion statistic = 0.87 (*p* = 0.61). β = regression coefficient on the log scale; Std. Error = standard error.

**Table 4:** Global Moran’s I Summary Statistics for ZCTA-Level Breast Cancer Screening Counts, Hillsborough County, Florida.

Statistic	Value	Interpretation
Moran’s Index	0.002727	Near zero
Expected Index	−0.019608	Baseline under randomness
Variance	0.004704	—
z-score	0.325651	Not significant
*p*-value	0.744688	Fail to reject H_0_
Conceptualization	Inverse Distance	—
Distance Method	Euclidean	—

Note. Analysis performed in ArcGIS Pro. Input field: CASES. Conceptualization: Inverse Distance; Distance Method: Euclidean. H_0_: Spatial pattern is randomly distributed.

**Table 5:** Bayesian Poisson Regression Posterior Estimates, Hillsborough County, Florida (*n* = 55 ZCTAs; 4 chains; 8000 post-warmup draws).

Parameter	Estimate	Est. Error	l-95% CI	u-95% CI	R̂	Bulk ESS
Intercept	3.63	0.02	3.59	3.68	1.00	1819
Female_Population_White	0.22	0.03	0.15	0.28	1.00	1041
Female_Black_African_American	0.13	0.03	0.06	0.19	1.00	889
Female_Population_Hispanic_Latino	0.09	0.05	0.00	0.19	1.00	1166
Female_Population_Asian	0.02	0.02	−0.02	0.06	1.00	1105
Female_Population_Am_Indian_Alaska	0.02	0.03	−0.04	0.08	1.00	1581
Female_40_49	0.04	0.06	−0.08	0.14	1.00	913
Female_50_64	−0.01	0.06	−0.12	0.10	1.00	1041
Female_65	0.02	0.03	−0.04	0.07	1.00	1244
Median_Income	0.00	0.04	−0.07	0.07	1.00	1391
Female_Uninsured	0.05	0.06	−0.07	0.16	1.00	881

Note. CrI = credible interval. R̂ = potential scale reduction factor; values of 1.00 indicate full convergence. Bulk ESS = bulk effective sample size. All covariates were standardized before estimation (df_std).

**Table 6:** Model Fit Comparison: Bayesian Poisson vs. Bayesian Negative Binomial Regression.

Metric	Bayesian Poisson	Bayesian Neg. Binomial	Preferred Model	Direction
Bayesian R^2^	0.91 (SE: 0.01)	0.82 (SE: 0.04)	Poisson	Higher = better
RMSE	5.40	8.31	Poisson	Lower = better
MBE	0.02	1.40	Poisson	Lower = better
DIC	367.2	409.4	Poisson	Lower = better

Note. DIC = Deviance Information Criterion; lower values indicate better model fit. RMSE = Root Mean Square Error; MBE = Mean Bias Error. R^2^ is reported as the posterior mean with standard error.

## Data Availability

The data analyzed in this study are derived from publicly available U.S. Census Bureau sources and the Florida Cancer Data System (https://fcds.med.miami.edu, accessed on 1 January 2024). Aggregated ZCTA-level datasets, model specifications, and R code supporting the reported results are available from the corresponding author upon reasonable request.
